# The Antagonism of 5-HT6 Receptor Attenuates Current-Induced Spikes and Improves Long-Term Potentiation *via* the Regulation of M-Currents in a Pilocarpine-Induced Epilepsy Model

**DOI:** 10.3389/fphar.2020.00475

**Published:** 2020-04-28

**Authors:** Chaofeng Zhu, Rong Lin, Changyun Liu, Mingzhu Huang, Feng Lin, Gan Zhang, Yuying Zhang, Junjie Miao, Wanhui Lin, Huapin Huang

**Affiliations:** ^1^Department of Neurology, Fujian Medical University Union Hospital, Fuzhou, China; ^2^Department of Electrophysiology, Fujian Key Laboratory of Molecular Neurology, Fuzhou, China; ^3^Department of Geriatrics, Fujian Medical University Union Hospital, Fuzhou, China

**Keywords:** 5-HT6R, KCNQ2/3, M-current, long-term potentiation, epilepsy

## Abstract

Recent studies have documented that reduced M-current promotes epileptogenesis and attenuates synaptic remodeling. Neurite growth is closely related to the level of 5-HT6 receptor (5-HT6R) in the central nervous system. However, little research is available regarding the relation between 5-HT6R and M-current and the role of 5-HT6R in M-current regulation. Herein, we found that the expression of 5-HT6R was notably increased and the expression of KNCQ2/3, the main components of the M channel, was decreased in a time-dependent manner in pilocarpine-induced chronic epileptic hippocampus. Interestingly, antagonism of 5-HT6R by SB271046 upregulated the expression of KCNQ2 but not KCNQ3. SB271046 greatly alleviated excitatory/inhibitory imbalance and improved the impaired LTP in the chronic epileptic hippocampus. Further mechanism exploration revealed that the above effects of SB271046 can be reversed by the M-channel inhibitor XE991, which also confirmed that SB271046 can indeed improve abnormal M current. These data indicate that the antagonism of 5-HT6R may decrease the excitability of hippocampal pyramidal neurons in chronic epileptic rats and improve the impaired long-term potentiation by upregulating the expression of KCNQ2 in the M-channel.

## Introduction

Cognitive impairment is common in temporal lobe epilepsy (TLE) (Huang et al., 2019), and the typical characteristic of pathological changes in TLE is hippocampal sclerosis, including neuronal loss in the CA3/CA1/DG region, glial proliferation, and abnormal mossy fiber sprouting (Schmeiser et al., 2017; Prada Jardim et al., 2018; Tai et al., 2018). Long-term potentiation (LTP), a neuronal model of neuroplasticity, is the physiological basis of memory formation (Kullmann and Lamsa, 2007; Bannerman et al., 2014). There are several forms of LTP, including the typical form, in which the recording electrode is on CA1 and the stimulating electrode is on CA3. Neuronal loss and lesions in chronic spontaneous recurrent seizures (SRSs) occur in CA3/CA1/DG over the course of TLE (Sloviter, 1994; Bannerman et al., 2014). Therefore, the loss of neurons in TLE is spread and brings about hippocampus-dependent learning and memory impairment, which could be detected *via* LTP. However, few studies have reported how TLE disturbs LTP.

The serotonin (5-hydroxytryptamine) receptor 5-HT6 (5-HT6R) is exclusively expressed in both the developing and the mature nervous systems (Roberts et al., 2002; Dayer et al., 2015; Helboe et al., 2015). It is highly expressed in mnemonic regions such as the olfactory tubercle, striatum, nucleus accumbens, and hippocampus (De Deurwaerdere et al., 2020). Studies have proven that it plays important roles in neuronal migration, neuronal differentiation, and neurite growth (Roberts et al., 2002; Dayer et al., 2015; De Deurwaerdere et al., 2020). Considering its particular distribution, 5-HT6R has recently been targeted to alleviate cognitive impairment, especially hippocampus-dependent learning and memory impairment (Seo and Tsai, 2014; Dayer et al., 2015; Karila et al., 2015; Aparicio-Nava et al., 2019). Interestingly, 5-HT6R blockers have antiepileptic effects. As early as 2000, 5-HT6R antagonists were demonstrated to increase the threshold of seizures in the maximal electroshock seizure test (Routledge et al., 2000). In 2015, increased 5-HT6R expression was found both in human TLE and kainic-acid-induced epileptic mouse models, and it was shown that 5-HT6R antagonists could alleviate seizures in the model (Wang et al., 2015). Our previous studies have demonstrated that the inactivation of 5-HT6R with SB271046 not only attenuates spontaneous recurrent seizures but also improves learning and memory performance in rats with pilocarpine-induced epilepsy (Lin et al., 2017; Liu et al., 2019). Therefore, 5-HT6R is theoretically considered to be a target for precognitive effects in an epileptic rodent model. However, the mechanism remains to be further explored.

Accumulating evidence has shown that 5-HT6R has a high level of constitutive activity established for cultured rodent brains, cultured neurons, and cell lines such as HEK-293 (). The activity of 5-HT6R was preliminarily considered to depend on its coupling to Gs, which is involved in alternative signaling mechanisms, including the rapamycin (mTOR) pathway, RhoA-dependent pathway, Fyn-ERK1/2 pathway, and p-c-Jun-Jab-1 pathway (Chaumont-Dubel et al., 2019). Further studies proved that 5-HT6R recruited cyclin-dependent kinase 5 (Cdk5) and is activated by the Cdc42-dependent pathway (Pujol et al., 2020). Both of the above pathways are related to neuronal migration, neurite growth, and synaptogenesis. 5-HT6R is positively linked to adenylate cyclase (AC) to increase cAMP formation, which is related to inducing LTP (Otmakhova et al., 2000). Several studies have shown that 5-HT6R antagonists increase LTP in cAlzheimer's disease/Vascular dementia (Aparicio-Nava et al., 2019; Grychowska et al., 2019; Rychtyk et al., 2019; Shahidi et al., 2019). Notably, a few studies demonstrated that 5-HT6R agonists also showed precognitive effects in cognitive disorders (Kendall et al., 2011; Vanda et al., 2018; Rychtyk et al., 2019). The contradictory mechanism remains unclear. Published data indicate that the regulatory role of 5-HT6R depends on the distribution of neurotransmitter systems. If an agonist activates 5-HT6R located directly on cholinergic and/or glutamatergic neurons, it may lead to an increase in cholinergic and glutamatergic transmission, while 5-HT6R antagonists act *via* GABAergic interneurons. Previous studies have indicated little expression of 5-HT6R on cholinergic and glutamatergic neurons in healthy conditions (Woolley et al., 2004). However, how 5-HT6R is expressed and how its ligands act in an epileptic model remain unclear. The KCNQ2/3 channel is the main isoform of the voltage-gated potassium channel and is expressed in both pyramidal neurons and interneurons (Soh et al., 2014a), the latter being fundamental regulators of normal brain activity. The KCNQ2/3 channel underlies the neuronal M-current. A dysfunction of the KCNQ2/3 channel in forming the M-current has been shown to be responsible for multiple pediatric epileptic disorders, such as benign neonatal familial convulsion (BNFC) and early-onset neonatal epileptic encephalopathy (Surti and Jan, 2005). Several studies have demonstrated that the suppression of the M-current has antiepileptic effects in rodent models (Kay et al., 2015; Greene et al., 2018). In pyramidal neurons, the KCNQ2/3 channel primarily controls spike frequency adaptation, preventing quiescence-period neurons from entering a brief chain of activities (Rodier et al., 2018; Carver and Shapiro, 2019). Other studies have found that the deletion of either KCNQ2/3 or KCNQ2 channels from PV+ interneurons can induce elevated homeostatic potentiation of fast excitatory transmission in pyramidal neurons (Soh et al., 2018) and that decreased expression of KCNQ2 and KCNQ3 in a stress model can impair spatial learning and memory and LTP in the hippocampus (Petrovic et al., 2012; Li et al., 2014). KCNQ2/3 channels co-localize to the axon initial segment (AIS) in the hippocampus, indicating their role in action potential (AP) generation (Dai et al., 2013). Further functional studies demonstrated that the suppression of the M-current in CA1 neurons reduced the intrinsic subthreshold of theta resonance and augmented spikes after depolarization (Ford et al., 2004), inducing the burst firing mode of the hippocampus. Another behavior study showed that the M-current inhibitor XE991 could revert cognitive impairment in a rodent model (Fontan-Lozano et al., 2011). The above data indicate that the KNCQ2/3 channel plays an important role not only in network excitability but also in synaptic remodeling. Since the M-current has been proven to be a “clamper” of neuronal potentiation, we hypothesized that the M-current might be a key molecule mediating 5-HT6R ligand-induced changes in LTP in a chronic epileptic rodent model.

In the present study, acute and chronic epileptic models were established with Sprague-Dawley (SD) rats by pilocarpine injection. The pilocarpine-induced chronic epileptic rat model mimics the progress of human temporal lobe epilepsy (Kandratavicius et al., 2014). Patch-clamp was employed to detect the excitability of hippocampal pyramidal neurons, the M-current, and long-term potentiation (LTP) in the hippocampus. The 5-HT6 antagonist SB271046, agonist WAY181187, M-current-opening NEM, and M-current inhibitor XE991 were used to explore the mechanism of 5-HT6R ligand-induced changes in LTP *via* the M-current.

## Materials and Methods

### Animals

Adult male Sprague-Dawley rats (weighed 200–250g) were provided by the Experimental Animal Center of Fujian Medical University and housed in an SPF animal laboratory (at 22-24°C, on a 12-h light/dark cycle) with free access to food and water. The R5 rat herd cage was used (size: 545*395*200 mm), and rats were housed four or five per cage. The animals stayed in the laboratory for half an hour before the experiment to adapt to the laboratory environment. The present study was approved by the Fujian Medical University Animal Experimental Ethical Committee (No. FJMUIACUC 2019-0058). The relevant experimental protocols and procedures followed the Guidelines for the Care and Use of Laboratory Animals in Fujian Medical University.

### Differential Expression Analysis

The “LIMMA” package in R software was utilized to obtain differentially expressed genes (DEGs) among unaffected samples, epileptic samples, and SB271046-treated samples (10 mg/kg, i.p. for 3 consecutive days follow 30 days after seizure). p-value < 0.05 and |fold change (FC)| > 2 were set as restrictive conditions to identify DEGs. Moreover, Venn diagrams were produced by using online tools (http://bioinfogp.cnb.csic.es/tools/venny/index.html).

### Materials

SB271046 (Purity: ≥99% (HPLC); 5-Chloro-N-[4-methoxy-3-(1-piperazinyl) phenyl]-3-methyl-benzo[b]thiophen-2-sulfonamide hydrochloride; CAS Number: 209481-24-3), WAY181187, and XE991 were obtained from Tocris (Ellisville, MO, USA). The other drugs and reagents were purchased from Sigma (St. Louis, MO, USA). Stock solutions for the drugs mentioned above were DMSO or 0.9% saline solution. All of the drugs tested in electrophysiological recording, including SB-271046 (300nM), WAY-181187 (200nM), NEM (20μM), and XE991 (10μM), were dissolved in artificial cerebral spinal fluid (ACSF) and maintained in slice recording chambers by bath exchange for at least 20 min (West et al., 2009; Zhou et al., 2011).

### Pilocarpine-Induced SE and Treatment

Normal adult SD rats were treated as described (Glien et al., 2001; Sun et al., 2018). Briefly, rats were first intraperitoneally injected (i.p.) with lithium chloride (127 mg/kg, Sigma) and pilocarpine (30 mg/kg, Sigma) 16–18 h later. Atropine sulfate (1mg/kg) was administered 30 min before the pilocarpine injection to antagonize the peripheral cholinergic effect. In the absence of status epilepticus (SE) symptoms, the injection of pilocarpine (30 mg/kg) was repeated at an interval of 30 min. Animals were considered SE-kindled and selected for further analyses when they displayed epileptic symptoms that were classified as stage 4-5 SE (Racine, 1972) and sustained for more than 30 min. To terminate seizures, diazepam (10 mg/kg) was intraperitoneally injected into the animals. Out of the selected animals, 72 male SD rats were divided into six subgroups according to the time from SE onset, with the saline group as the control. They were sacrificed at different time points (30 min, 1 d, 4 d, 7 d, 14 d, 30 d) after SE onset, and their brains were removed for Western blotting (each group, n=4) to analyze the expression of 5-HT6R and KCNQ2/3. Thirty days after SE, the living rats were randomly divided into three groups: the Pilo group, receiving 0.9% saline (i.p.) (n=121); the Pilo+SB271046 group, receiving SB271046 (10 mg/kg, i.p.) for 3 consecutive days according to a previous study (Chen et al., 2018) (n=8); the Pilo+ WAY181187 group, receiving WAY181187 (17 mg/kg, i.p.) for 3 consecutive days (Carr et al., 2011) (n=8). For the saline group, rats were intraperitoneally injected with 0.9% saline solution (n=27). The hippocampus and prefrontal cortex from each group (n=8) were analyzed by Western blotting, and the remaining rats were randomly assigned into seven groups for electrophysiological recording:

- Saline group (n=21), with slices maintained in ACSF;
- Pilo group (n=20), with slices maintained in ACSF;
- Pilo+SB271046 group (n=20), with slices maintained in ACSF+SB-271046 (300 nM);
- Pilo+WAY181187 (n=21) group, with slices maintained in ACSF+WAY-181187 (200 nM);
- Pilo+NEM group (n=10), with slices maintained in ACSF+NEM (20 μM);
- Pilo+XE991 group (n=10), with slices maintained in ACSF+XE991 (10 μM);
- Pilo+SB271046+XE991 group (n=11), with slices maintained in ACSF+SB-271046 (300nM)+XE991 (10 μM).

## Electrophysiology

Rats received Demerol and were anesthetized with isoflurane for the preparation of hippocampal slices. The brains were excised rapidly, placed into ACSF and coronally cut into slices (400 μm) using vibratome (Leica VT1000S) in ice-cold oxygenated ACSF (95% O_2_/5% CO_2_), which contained NaCl (126 mM), NaHCO_3_ (18 mM), KCl (2.5 mM), NaH_2_PO_4_ (1.2 mM), CaCl_2_ (1.2 mM), MgCl_2_ (2.4 mM), and Glucose (11 mM) (pH 7.3, 325 mOsm/l). The rat brain atlas was used, and the cuts were made in Z coordinates. Slices were incubated in the ACSF at 32°C for 30 min and then at room temperature for over 30 min. Afterward, they were removed to the recording chambers for persistent perfusion (2 ml/min) with oxygenated ACSF.

For field excitatory postsynaptic potential (fEPSP) recording, the external solution was adapted from Tengfei Ma et al. (2018), and the microelectrode was filled with ACSF (3–6 M resistance). fEPSPs were recorded using a stimulation protocol of 0.1 Hz with an increasing stimulation intensity from 0 to 100 μA at an increment of 10 μA and were used for input-output analysis. Paired-pulse stimulations were delivered at inter-stimulus intervals (ISI) of 10, 30, 50, 100, 200, and 500 ms. Paired-pulse ratios (PPRs) were calculated by dividing the magnitude of the response to the 2nd stimulation by the magnitude of the response to the 1st stimulation of the paired pulses. For LTP, a 20-min fEPSP baseline (measured as 30% of the maximum response) was recorded with the protocol of a stimulus pulse width of 0.1 ms at 10 Hz before the high-frequency stimulation (HFS, 100 Hz, 1s) was applied two times. Following HFS, responses to the test stimulation were recorded for another 60 min.

For whole-cell recording of spontaneous excitatory postsynaptic currents (sEPSCs) and spontaneous inhibitory postsynaptic currents (sIPSCs), the pipette solution contained CsCH_3_SO_3_ (140 mM), MgCl_2_ (2 mM), TEA-Cl (5 mM), HEPES (10 mM), EGTA (1 mM), Mg-ATP (2.5 mM), and Na_2_-GTP (0.3 mM), with pH value adjusted to 7.2–7.4 with CsOH. Spontaneous EPSCs (sEPSCs) were recorded at a holding potential of -70 mV, and spontaneous IPSCs (sIPSCs) were recorded at a holding potential of 0 mV. For evoked spikes recording, the brain slices were perfused with ACSF and the glass pipette was filled with a solution containing K-gluconate (140 mM), MgCl_2_ (2 mM), CaCl_2_ (0.1 mM), HEPES (10 mM), EGTA (1.1 mM), Na2-GTP (0.3 mM), and Na_2_-ATP (pH =7.25, adjusted with KOH).

For recordings of M-currents, the method was adapted from the reported literature (Zhou et al., 2011). Briefly, pipettes were filled with an internal solution containing KCl (120 mM), MgCl_2_ (1.5 mM), Na_2_-ATP (2 mM), CaCl_2_ (1.0 mM), BAPTA (1.0 mM), and HEPES (10 mM). The external solution contained NaCl (115 mM), KCl (2.5 mM), MgCl_2_ (2.0 mM), HEPES (10 mM), BAPTA (0.1 mM), Glucose (10 mM), and tetrodotoxin (0.1 μM). The membrane potential was held at -30 mV for M-current activation. A de-activation protocol was applied. Serial resistance was maintained between 20 and 30 MΩ and was 70% compensated. Recordings were filtered at 3 kHz and sampled at 10 kHz.

### Western Blotting

Proteins were extracted from the hippocampus of rats, separated by SDS-PAGE, and then transferred onto PVDF membranes. After being blocked with 5% skim milk in PBST at room temperature for 2 h, the membranes were incubated with primary antibodies overnight at 4°C: 5-HT6R (1:1000, Sigma), KCNQ2 (1:800, Abcam), KCNQ3 (1:800, Abcam), and GAPDH (1:1000, Shanghai, China). The primary antibodies against KCNQ2, KCNQ3, GAPDH originated from rabbit and were polyclonal, while the primary antibody against 5-HT6R originated from rabbit and was monoclonal. Next, the membranes were washed using PBST and incubated with horseradish peroxidase (HRP)-conjugated secondary antibody at room temperature for 60 min. The specific secondary antibodies against 5-HTR6 (1:1000) and KCNQ2 (1:2000) and KCNQ3 (1:2000) and GADPH (1:2000) all originated from goat. The signals were detected using an electrochemiluminescence (ECL) kit (Millipore, St. Charles, MO, United States) and a Bio-Rad electrophoresis image analyzer.

### Statistical Analysis

All data are represented as mean ± SEM. Statistical analyses were performed with Graph Pad Prism6, SPSS20.0, Clampfit software. Student's t-tests were performed for two-group comparisons. One-way ANOVA and two-way ANOVA with Bonferroni tests were conducted for experiments with more than two groups. A value of *p* < 0.05 was considered statistically significant.

## Results

### Identification of Significant Differentially Expressed Genes (DEGs) in Pilocarpine-Induced Chronic Epileptic Hippocampus After SB271046 Treatment

**Figure 1:**
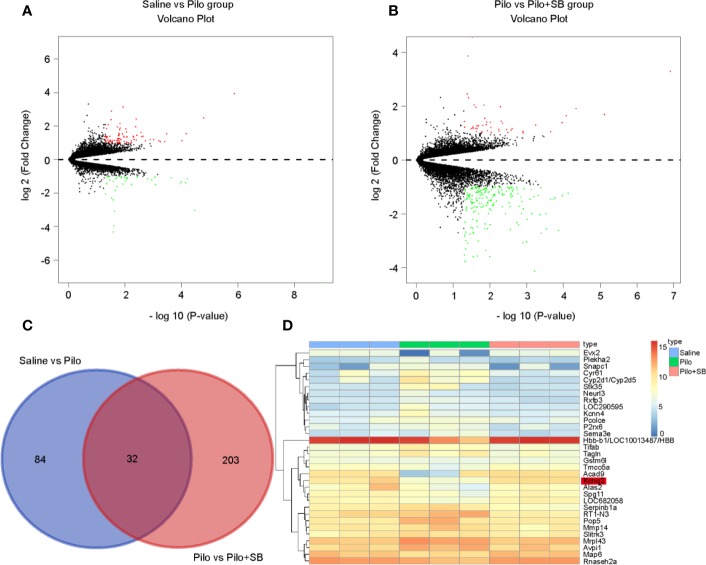
Thirty-two significant differentially expressed genes (DEGs) were identified in epilepsy after SB271046 treatment. **(A)** Volcano plot showing the DEGs identified between the Saline and Pilo groups. **(B)** Volcano plot showing the DEGs identified between the Pilo and SB271046 treatment groups (each group n=3). The x-axis represents log-transformed P-value, and the y-axis indicates the mean expression differences of genes. Note: The two volcano plots show all of the DEGs; the black dots represent genes that are not differentially expressed, and the green dots and red dots represent the downregulated and upregulated genes, respectively. |log2FC| > 1 and p-value < 0.05 were set as the cut-off criteria. **(C)** The intersection of DEGs of the Saline vs Pilo group and Pilo vs SB271046 treatment group. **(D)** Heatmap of differential expressed genes between the Saline, Pilo and SB271046 treatment groups.

As depicted in **Figure 1A**, a volcano plot showed 80 upregulated (shown as red dots) and 36 downregulated (shown as green dots) DEGs between the Saline and Pilo group. Similarly, there were 45 upregulated and 190 downregulated DEGs between the Pilo and the Pilo+SB group (**Figure 1B**). Using |log2Fold change| > 1 and p-value < 0.05 as the threshold, as shown in **Figure 1C**, there were 116 DEGs between the Saline and Pilo group and 235 DEGs between the Pilo and the Pilo+SB group. The heatmap demonstrated 32 over-lapping genes, as shown in **Figure 1D**. Among them, KCNQ2 was one of the overlapping genes that were downregulated obviously in the Pilo group (shown in light blue) but upregulated distinctively after SB treatment (shown in light orange).

### Increased Expression of 5-HT6R and Decreased Expression of KCNQ2/3 in Rats With Pilocarpine-Induced Chronic Epilepsy

**Figure 2:**
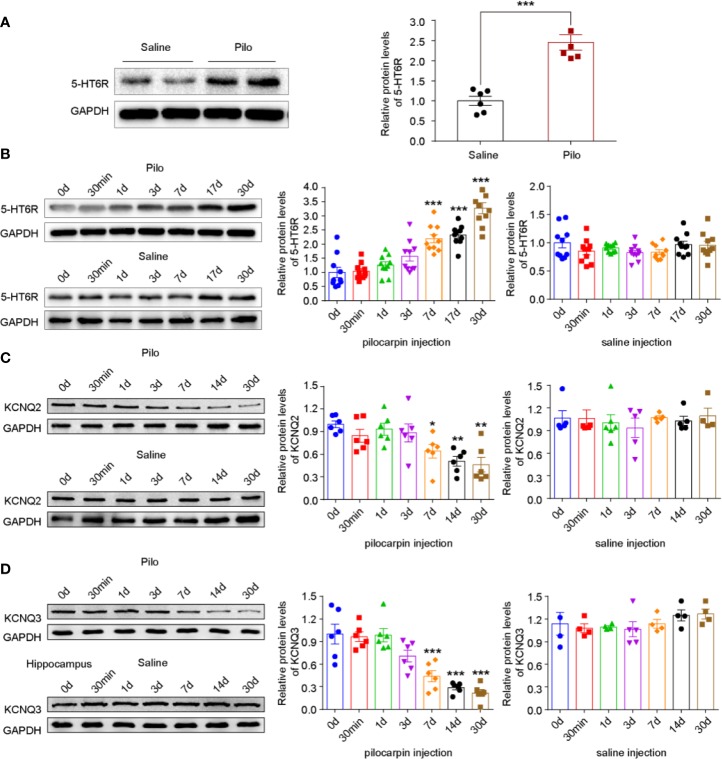
Pilocarpine-induced SE differentially affects 5-HT6R and KNCQ2/3 expression levels in a time-dependant manner. **(A)** The expression of 5-HT6R in chronic epileptic rats. It was significantly increased in the hippocampus when compared with the Saline group (Saline group n= 4; Pilo group n= 4; t (10) = -6.424, *p*=0.0001). Statistical comparisons were analyzed by two-tailed unpaired Student's t-test (*p < 0.05, **p < 0.01, ***p < 0.001). **(B)** The expression of 5-HT6R in the hippocampus after pilocarpine-kindled status epilepticus (SE). No change was evident across the timepoints after SE in the Saline group (n = 5 for each subgroup), while significant changes were observed in the Pilo subgroups (SE0d, SE30min, SE1d, SE3d, SE7d, SE14d, and SE30d), F (6,63) =30.753, *p* < 0.001). When compared with the baseline (before SE, SE0d), the expression of 5-HT6R increased notably in the SE7d, SE14d, and SE30d groups (*p* < 0.001, *p* < 0.001, and *p* < 0.001, respectively). Statistical comparisons were performed with a one-way analysis of variance (ANOVA, *p <*0.05) followed by the Bonferroni post-hoc test (*p < 0.05, **p < 0.01, **p < 0.001). **(C)** The expression of KCNQ2 in the hippocampus after pilocarpine-kindled status epilepticus (SE). Across the timepoints after SE, no change was evident in the Saline group (n = 5 for each sub-group), while significant changes were observed in the Pilo subgroups (SE0d, SE30min, SE1d, SE3d, SE7d, SE14d, and SE30d), F (6,35) =7.426, *p* < 0.001). When compared with the baseline (before SE, SE0d), the expression of KCNQ2 increased notably in the SE7d, SE14d, and SE28d groups (*p* = 0.0258, *p* = 0.0019, and *p*= 0.0010, respectively). Statistical comparisons were performed with one-way analysis of variance (ANOVA, *p <*0.05) followed by the Bonferroni post-hoc test (*: *p <* 0.05, **p < 0.01, ***p < 0.001). **(D)** The expression of KCNQ3 in the hippocampus after pilocarpine-kindled status epilepticus (SE). Across the timepoints after SE, no change was evident in the Saline group (n = 5 for each sub-group), while significant changes were observed in the Pilo subgroups (SE0d, SE30min, SE1d, SE3d, SE7d, SE14d, and SE30d), F (6,35) =18.793, *p* < 0.001);. When compared with the baseline (before SE, SE0d), the expression of KCNQ3 increased notably in the SE7d, SE14d, and SE28d groups (*p*=0.0003, *p* < 0.001, and *p* < 0.001, respectively). Statistical comparisons were performed with one-way analysis of variance (ANOVA, *p <* 0.05) followed by the Bonferroni post-hoc test (*p < 0.05, **p < 0.01, ***p < 0.001).

To explore the potential role of 5-HT6R and KCNQ2/3 in epilepsy, an epileptic rat model was induced by pilocarpine and the proteins in the hippocampus of the rats with spontaneous recurrent seizures (SRSs) were analyzed *via* Western blotting. The level of 5-HT6R significantly increased in these chronic epileptic rats when compared with that of the controls, t (10) = -6.424, *p*=0.0001 (**Figure 2A**). Next, to observe the variation in 5-HT6R and KCNQ2/3 expression after pilocarpine-induced seizures, acute SE rats were included in the experiment. The level of 5-HT6R increased in a time-dependent manner and peaked in the chronic period (F (6,63) =30.753, *p* < 0.001) (**Figure 2B**). However, the level of both KCNQ2 and KCNQ3 in the hippocampus decreased gradually in a time-dependent manner and dropped to a minimum in the chronic period (F (6,35) =7.426, *p* < 0.001 and F (6,35) =18.793, *p* < 0.001, respectively) (**Figures 2C, D**).

### SB271046 Attenuates Hippocampal Neuronal Excitability and Enhances LTP

**Figure 3:**
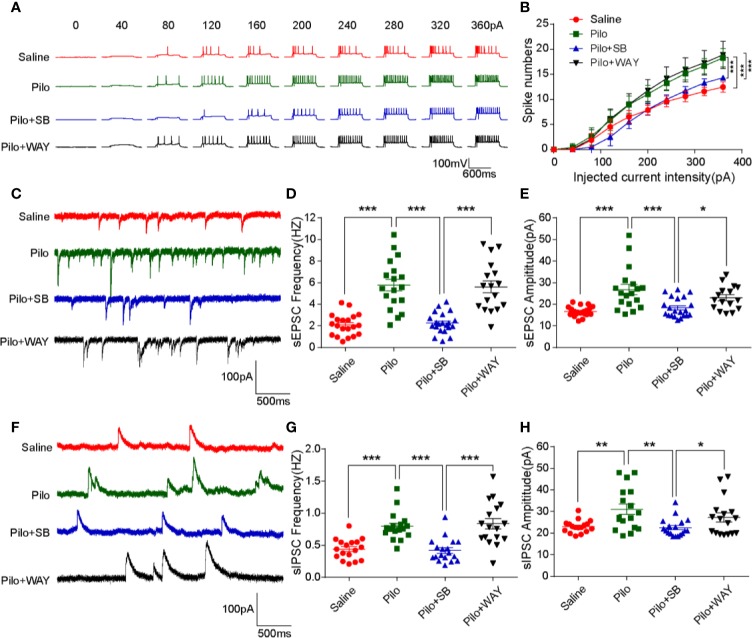
Antagonism of 5-HT6R with SB271046 inhibited hippocampal pyramidal neuronal excitability. **(A)** Representative traces of evoked action potential (APs) in response to various depolarization currents injected into individual hippocampal neurons. **(B)** Quantification of evoked action potentiation. The number of spikes in the Pilo group increased significantly when compared with the Saline group and decreased notably after SB271046 treatment, while WAY181187 treatment produced no significant change. For comparing: ANOVA, F (3,686) = 12.962, *p* < 0.001; for the Saline group (n=17/6) vs. the Pilo group (17/5): *p*= 0.0002; for the Pilo group vs. the Pilo+SB group (n=18/6), *p*= 0.0003; for the Pilo+SB group vs. the Pilo+WAY group (n=17/6), *p* < 0.001. Significance was determined using two-factor mixed ANOVA followed by the Bonferroni *post hoc* test. (*p < 0.05, **p < 0.01, ***p < 0.001). **(C)** Representative sEPSC traces of the four groups. **(D)** Statistic analysis of sEPSC frequency. For comparing sEPSC frequency: ANOVA, F (3,72) =26.655, *p* < 0.001; for the Saline group vs. the Pilo group: *p* < 0.001; for the Pilo group vs. the Pilo+SB group, *p* < 0.001; for the Pilo+SB group vs. the Pilo+WAY, *p* < 0.001. **(E)** Statistic analysis of sEPSC amplitude. For comparing sEPSC amplitude: ANOVA, F (3,71) = 10.750, *p* < 0.001; for the Saline group vs. the Pilo group: *p* < 0.001; for the Pilo group vs. the Pilo+SB group, *p* = 0.0003; for the Pilo+SB group vs. the Pilo+WAY, *p*= 0.0256. **(F)** Representative sIPSC traces of the four groups. **(G)** Statistic analysis of sIPSC frequency. For comparing sIPSC frequency: ANOVA, F (3,65) = 15.715, *p* < 0.001; for the Saline group vs. the Pilo group: *p*= 0.0002; for the Pilo group vs. the Pilo+SB group, *p*= 0.0001; the Pilo+SB group vs. the Pilo+WAY group, *p* < 0.001. **(H)** Statistic analysis of sIPSC amplitude. For comparing sIPSC amplitude, ANOVA, F (3,65) = 5.578, *p*= 0.0018; for the Saline group vs. the Pilo group: *p*= 0.0092; for the Pilo group vs. the Pilo+SB group, *p*= 0.0037; for the Pilo+SB group vs. Pilo+WAY, *p*= 0.0486. Statistical comparisons were performed with one-way analysis of variance (ANOVA, *p <* 0.05) followed by the Bonferroni post-hoc test (*p < 0.05, **p < 0.01, ***p < 0.001).

To evaluate the neuronal excitability, sEPSCs and sIPSCs of the pyramidal neurons from the CA1 region were recorded *via* voltage-clamp. The number of current-induced spikes significantly increased in the Pilo group when compared with that of the Saline group (*p*= 0.0002) and remarkably decreased in the Pilo+SB271046 group when compared with that of the Pilo group (*p*= 0.0003) (**Figures 3A, B**). WAY-181187 possesses high-affinity binding at the human 5-HT6 receptor and is profiled as a full receptor agonist. Both the frequency and amplitude of sEPSCs and sIPSCs increased notably in the Pilo group and significantly decreased in the Pilo+SB271046 group when compared with those of the Pilo group, while WAY181187 intervention produced no significant change (**Figures 3C–H**). These results indicate that pilocarpine-induced chronic epilepsy displays an increasing action potential (AP) and that 5-HT6R antagonist SB271046 can modulate the imbalance between excitatory and inhibitory neurotransmitters.

**Figure 4:**
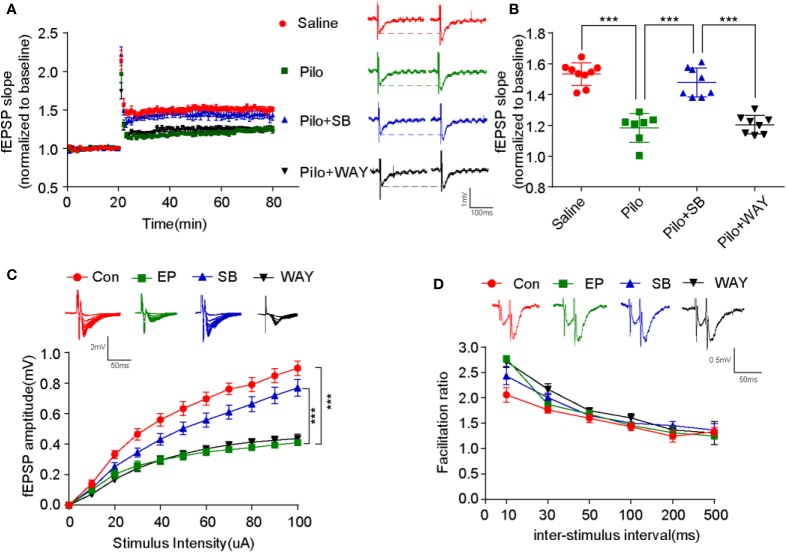
SB271046 improved hippocampal synaptic function in chronic epileptic rats. **(A)** Successful recording of LTP *via* patch-clamp. **(B)** Analysis of fEPSP during the last 10 minutes among the four groups. The potentiation in the Pilo group decreased significantly compared with that in the Saline group. It was rescued by SB271046 but not by WAY181187. For comparing: ANOVA, F (3,28) = 40.063, *p*= 0.0000; for the Saline group (n=9 from 5 rats) vs. the Pilo group (n=7/4, 7 from 4 rats): *p* < 0.001; for the Pilo group vs. the Pilo+SB group (n=8/4), *p* < 0.001; for the Pilo+SB group vs. the Pilo+WAY group (n=8/4), *p* < 0.001. Significance was determined by one-way ANOVA with Bonferroni *post hoc* analysis (***p < 0.001). **(C)** Input-output curve. The scatter plot was constructed by averaging three consecutive sweeps at a current interval of 10 μA. For comparing: ANOVA, F (3,346) = 50.277, *p* < 0.001; for the Saline group vs. the Pilo group: *p* < 0.001; for the Pilo group vs. the Pilo+SB group, *p* < 0.001; for the Pilo+SB group vs. the Pilo+WAY group, *p* < 0.001. Significance was determined using a two-factor mixed ANOVA with Bonferroni *post hoc* analysis. (***p < 0.001). **(D)** Paired-pulse facilitation ratios. No differences were observed among the four groups. F (3,128) = 1.361, *p* = 0.2576. Statistical comparisons were analyzed by two-factor mixed ANOVA with Bonferroni *post hoc* analysis.

Next, LTP from CA3-CA1 was recorded by high-frequency stimulation (HFS). The potentiation significantly decreased in the Pilo group when compared with that of the Saline group (p < 0.001). After SB271046 intervention, the potentiation greatly increased in the SB271046 group when compared with that of the Pilo group (*p* < 0.001) and WAY181187 intervention produced no change (**Figures 4A, B**). The amplitude of fEPSP gradually paralleled the increased stimulus intensity in the four groups, but the variation in the Pilo group decreased when compared with that of the Saline group (*p* < 0.001). The input-output response analysis suggests that chronic epileptic rats show decreased excitatory transmission (**Figure 4C**). However, the paired-pulse facilitation (PPF) ratios remained unchanged among the four groups (**Figure 4D**). These results suggest that 5-HT6R antagonist SB271046 improves synaptic transmission.

### SB271046 Rescues the Aberrant Neuronal M-Currents in Pilocarpine-Induced Chronic Epileptic Hippocampus

**Figure 5:**
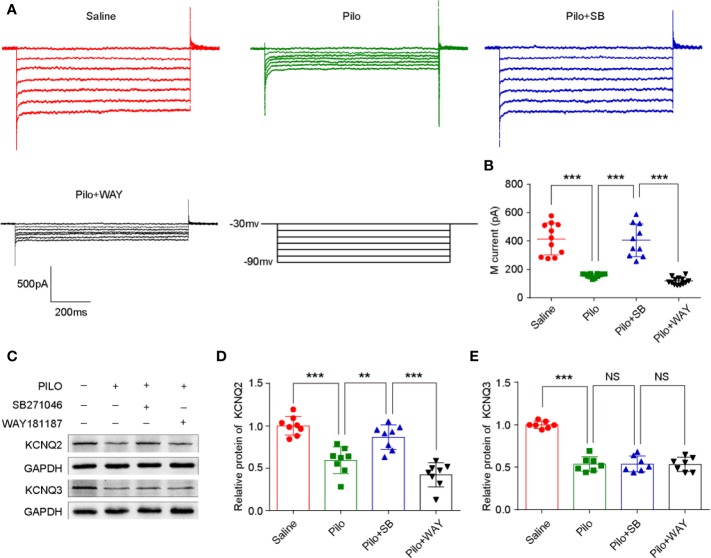
SB271046 rescued aberrant hippocampal neuronal M-current in chronic epileptic rats. **(A)** Hippocampal neuronal M-currents were activated at different voltage steps. **(B)** Analysis of the mean amplitude of M-currents at -50 mV in the hippocampal neurons. The amplitude of M-currents in the Pilo group decreased significantly when compared with that of the Saline group. SB271046 rescued the aberrant M-currents while WAY181187 intervention did not. For comparing: ANOVA, F (3,42) = 47.139, *p* < 0.001; for the Saline group (n =1 1/5) vs. the Pilo group (n = 12/6): *p* < 0.001; for the Pilo group vs. the Pilo+SB group (n = 10/5), *p* < 0.001; for the Pilo+SB group vs. the Pilo+WAY (n=13/6), *p* < 0.001. Statistical comparisons were performed with a one-way analysis of variance (ANOVA, *p* < 0.05) followed by the Bonferroni post-hoc test (**p < 0.01, ***p < 0.001). **(C)** Representative Western blot images of KCNQ2/3 and GADPH. **(D)** Statistic analysis of KCNQ2 protein expression. SB271046 increased the level of KCNQ2 in pilocarpine-induced chronic epileptic hippocampus. Statistical comparisons were performed with one-way ANOVA followed by the Bonferroni post-hoc test (**p < 0.01, ***p < 0.001). For comparing: ANOVA, F (3,28) = 27.559, *p* < 0.001; for the Saline group vs. the Pilo group: *p* < 0.001; for the Pilo group vs. the Pilo+SB group, *p*= 0.0033; for the Pilo+SB group vs. the Pilo+WAY, *p* < 0.001, n = 4 for each group. **(E)** Statistic analysis of KCNQ3 protein expression. Neither SB271046 nor WAY181187 treatment affected the level of KCNQ3 in pilocarpine-induced chronic epileptic hippocampus. For comparing: ANOVA, F (3,24) = 60.669, *p* < 0.001; for the Saline group vs. the Pilo group: *p* < 0.001; for the Pilo group vs. the Pilo+SB group, *p*= 0.9541; for the Pilo+SB group vs. the Pilo+WAY, *p* = 0.9265. Statistical comparisons were performed with one-way ANOVA followed by the Bonferroni post-hoc test (**p < 0.01, ***p < 0.001 NS, not significant).

M-currents from CA1 pyramidal neurons were activated or inactivated *via* the whole-cell clamp method according to a previous study (Zhou et al., 2011). A striking difference in the M-channel activity was evident among the four groups (F (3,42) = 47.139, *p* < 0.001). The magnitude of M-currents in the Pilo group significantly decreased when compared with that of the Saline group (*p* < 0.001). After SB271046 treatment, the magnitude of M-currents was significantly augmented compared with that of the Pilo group (*p* < 0.001), whereas WAY181187 intervention produced decreased effects (**Figures 5A, B**).

### Inactivated 5-HT6R Improves Synaptic Function *via* Regulating the M-Channel

**Figure 6:**
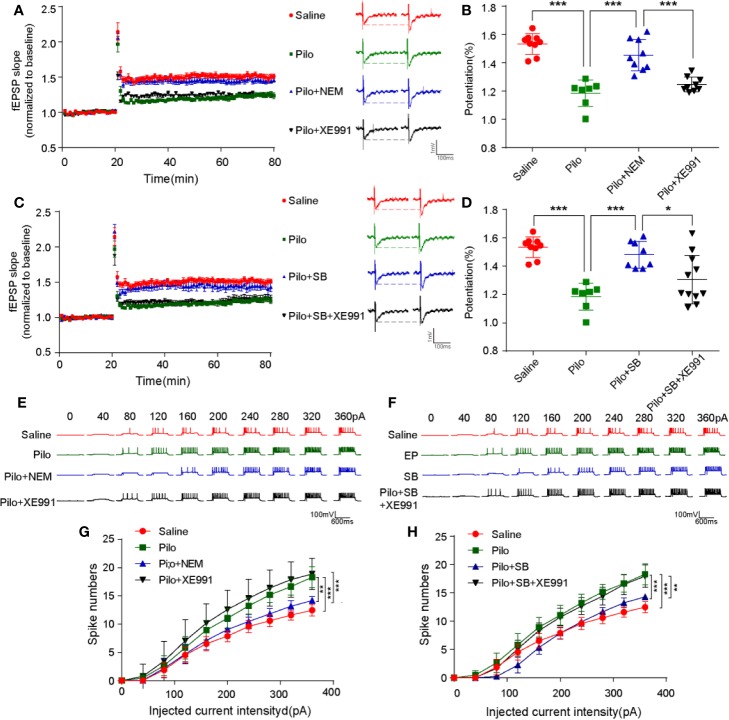
SB271046 rescued synaptic functions by regulating M-channel in chronic epileptic rats. **(A, C)** LTP recordings from epileptic hippocampal slices after the treatment of 5-HT6R ligands and M-channel ligands. **(B, D)** Analysis of potentiation among the four groups. The potentiation of LTP in the Pilo group was notably upregulated compared with that in the Saline group, which was reversed by both NEM and SB271046 but not XE991. Moreover, the effect of SB271046 was reversed by XE991. For comparing: ANOVA, F(3,30) = 31.466, *p* < 0.001; for the Saline group (n=9/5) vs. the Pilo group (n=7/4): *p* < 0.001; for the Pilo group vs. the Pilo+NEM group (n=9/5), *p*= 0.0000; for the Pilo+NEM group vs. the Pilo+XE991 group (n=9/5), *p* < 0.001. For comparing: ANOVA, F(3,31) = 14.287, *p* < 0.001; for the Pilo group vs. the Pilo+SB group, *p*= 0.0003; for the Pilo+SB271046 group vs. the Pilo++SB271046+XE991 group (n=11/5), *p*= 0.0222. Statistical comparisons were performed with one-way ANOVA followed by the Bonferroni post-hoc test (*p < 0.05, **p < 0.01, ***p < 0.001). **(E, G)** Representative traces of evoked action potential in response to various depolarization currents injected into individual hippocampal neurons. **(F, H)** Quantification of evoked action potentials. (Saline group n=17/6; Pilo group n=16/6; Pilo+NEM group n=17/6; Pilo+XE991 group n=17/6; Pilo+SB271046 group n=17/6; Pilo+SB271046+XE991 group n=17/6). Significance was determined using two-factor mixed ANOVA followed by the Bonferroni post-hoc test. (*p < 0.05, **p < 0.01, ***p < 0.001). ANOVA, F (3,676) = 13.551, *p* < 0.001; for the Saline group vs. the Pilo group: *p*= 0.0002; for the Pilo group vs. the Pilo+NEM group, *p* = 0.0017; for the Pilo+NEM group vs. the Pilo+XE991 group, *p* = 0.0001. For comparison: ANOVA, F(3,676) = 10.465, *p* < 0.001; for the Pilo group vs. the Pilo+SB271046 group, *p* = 0.0001; for the Pilo+SB271046 group vs. the Pilo++SB271046+XE991 group, *p*= 0.0024. ‘n' designates the number of cells followed by number of animals. Data in summary graphs are represented as mean ± SEM.

N-ethylmaleimide (NEM), a cysteine-modifying reagent, was found to strongly augment M-currents by increasing their maximal open probability by alkylation of a cysteine residue in the channels' C terminus without affecting surface expression. The hippocampal neuronal current-induced spikes increased notably in the Pilo group compared with the Saline group (*p*= 0.0002), while inactivating 5-HT6R with SB271046 or opening M-channel *via* NEM decreased the number of spikes significantly (*p*= 0.0001, *p*= 0.0017, respectively). The effect was blocked when M-channel inhibitor XE991 was administered to the chronic epileptic hippocampal slices (**Figures 6E–H**).

Furthermore, in the Pilo group, the LTP of the hippocampus from CA3-CA1 was significantly attenuated (*p* < 0.001) but was rescued by SB271046 or NEM intervention (*p*= 0.0003, *p*= 0.0000, respectively). The treatment with M-channel inhibitor XE991 produced no change. The effect of SB271046 was blocked when XE991 was added to chronic epileptic hippocampal slices (*p*= 0.0222) (**Figures 6A–D**).

### Inactivated 5-HTR6 Upregulates the Expression of KNCQ2 But Not KNCQ3

As M-channel is composed of KCNQ2/3, the expression of KCNQ2/3 in the hippocampus was further analyzed by Western blotting. Notably, the level of both KCNQ2 and KCNQ3 in the Pilo group significantly decreased when compared with that in the Saline group. After the administration of SB271046, the expression of KCNQ2, but not KCNQ3, was markedly upregulated when compared with that of the Pilo group (**Figures 5C–E**).

## Discussion

Our current study demonstrated that SRSs increased the protein expression of 5-HT6R and decreased both the RNA of KCNQ2 and protein of KCNQ2 in hippocampus. We found that, in the pilocarpine-induced epileptic rat model, SB271046 rescued the aberrant M-current, reduced the excitability of the hippocampal pyramidal neurons, and improved the impaired LTP, which were reversed by XE991, which selectively inhibits M-current. These findings indicate that the antagonism of 5-HT6R may improve LTP and alleviate the excitatory/inhibitory imbalance in the hippocampal neurons by regulating the M-channel.

In line with previous studies, the protein expression of 5-HT6R was upregulated in the epileptic brain, especially in the chronic model (Wang et al., 2015; Liu et al., 2019). Pilocarpine-induced chronic epileptic rat model mimics the progress of human temporal lobe epilepsy (Kandratavicius et al., 2014). It usually involves an acute period (< 24 hours, status epilepticus), a silent period (> 24 hours, < 7 days, no seizures, no food or water, no movement), and a chronic period (> 7 days, SRSs). In our present study, the expression of 5-HT6R in hippocampus increased gradually during the silent period and peaked in the chronic period. These data suggest that spontaneous recurrent seizures may induce overexpression of 5-HT6R in hippocampus, which plays an important role in epileptogenesis. Combined with the results of RNA-sequencing and Western blotting, we speculate that SRSs may reduce the expression of 5-HT6R protein by affecting the translation level of 5-HT6R. In the literature, few studies have focused on whether 5-HT6R is involved in epilepsy. Previous studies reported that 5-HT6R ligands regulate the imbalance between exciting and inhibiting neurotransmitters (Karila et al., 2015). A recent study found that a 5-HT6R antagonist may modulate seizure activity (Wang et al., 2015). However, the neuronal activity has not been examined.

In our present study, spontaneous EPSC and IPSC were both detected in chronic epileptic hippocampal slices, the former mainly produced by the excitatory neurotransmitter receptors, such as NMDA-R/AMPA-R, and the latter by the inhibiting neurotransmitter receptor, such as GABA-R. Other studies report that the frequency of sEPSCs increases with or without decreased frequency of sIPSCs (Soh et al., 2014b). In this work, the frequency and amplitude of both sEPSCs and sIPSCs were markedly increased in the pyramid cells of the pilocarpine-induced chronic epileptic rat model. The cells also exhibited an increased number of current-induced spikes, further indicative of a shift towards hyperexcitability. Moreover, the antagonism of 5-HT6R by SB271046 decreased the frequency and amplitude of sEPSCs, sIPSCs, and action potentiation. These results indicate that 5-HT6R is overexpressed in the pilocarpine-induced chronic epileptic hippocampus and that the antagonism of 5-HT6R by SB271046 can modulate the imbalance between excitatory and inhibitory neurotransmitters.

We further explored the mechanism of the observed effect of 5-HT6R on epilepsy. Interestingly, the gene screening spectrum showed that antagonism of 5-HT6R upregulated the expression of KCNQ2 in pilocarpine-induced chronic epileptic hippocampus. It has been reported that the loss of KCNQ2 or KCNQ3 function induces neuronal hyperexcitability and that genetic mutations in KCNQ2/3 are responsible for multiple pediatric diseases, especially during infancy (Surti and Jan, 2005). Existing studies have probed into the role of KCNQ2/3 in the developing brain. Our present study investigated the role of KCNQ2/3 in the developed or mature brain. In our experiment, M-current was aberrant in the chronic epileptic slices, indicating a malfunction but not a loss of M-current. It is known that malfunctioning of the potassium channels constituted by KV7.2 and KV7.3 may lead to abnormality of the action potential (Fisher et al., 2005). The M-channel is composed of KV7.2 and KV7.3 subunits and plays an important role in the pathogenesis of epileptic seizure. The structures of voltage-gated potassium channels encoded by KCNQ family genes are quite similar and are tetramers composed of four identical or different α subunits. The pathogenic genes of KCNQ2 and KCNQ3 are located on chromosomes 20q13.3 and 8q24, respectively.

Ezogabine/Retigabine, targeting the M-channel, has been found to effectively control seizures in both animal and clinical tests (Blackburn-Munro et al., 2005; Gunthorpe et al., 2012). Moreover, between KCNQ2 and KCNQ3, KCNQ2 mutations are more common in neonatal or infantile seizures (Large et al., 2012). In this study, both KCNQ2 and KCNQ3 proteins were downregulated in the chronic epileptic hippocampus, which seems to be associated with SRSs. The SB271046-induced antagonism of 5-HT6R rescued the abnormal neuronal M-current, which was effectively reversed in response to M-channel inhibitor XE991. Interestingly, SB271046 upregulated the expression of KCNQ2 but not KCNQ3, which was consistent with the gene screening spectrum. Combined with the results of RNA-sequencing and Western blotting, we speculate that SB271046 can regulate the expression of KCNQ2 protein by affecting the transcription and translation of KCNQ2. The above data indicate that the activity of 5-HT6R may modulate the expression and function of KCNQ2 protein. It has been reported that the KCNQ2 subunit plays a more important role in forming the M-channel and producing M-current. However, it remains to be further explored whether and how the activity of 5-HT6R selectively modulates KCNQ2-induced M-current.

Furthermore, 5-HT6R has been reported to affect spatial learning and memory, which primarily relies on the function of the hippocampus (Dayer et al., 2015). However, it remains controversial whether the improved cognition is attributable to 5-HT6R antagonists or agonists (Karila et al., 2015; Asselot et al., 2017). In the current study, we found that SB271046 (a 5-HT6R antagonism) and not WAY181187 (a 5-HT6R agonist) improved the impaired hippocampal LTP in the chronic epileptic rats. Many reports have shown that the dysfunction of KCNQ/Kv7 channels is related to cognitive impairment. The reduction of KCNQ/Kv7 channels can mediate age-dependent memory decline (Wang et al., 2011; Cavaliere et al., 2013b). The M-channel plays an important role in regulating neuronal excitability, spike generation, hippocampal theta oscillation, and neurotransmitter release (Hu et al., 2002; Yue, 2004; Peters et al., 2005; Vervaeke et al., 2006), and suppressed M-currents in mice can impair the hippocampus-dependent spatial memory (Peters et al., 2005). Thus, it is evident that KCNQ/Kv7 channels are involved in synaptic plasticity, learning, and memory. Our current data reinforce the notion that the activation of functional M-channels by NEM may facilitate the enhancement of the hippocampal synaptic plasticity in pilocarpine-induced epileptic rats. In addition, 5-HT has been shown to inhibit M-currents in mammalian neurons (Roepke et al., 2012). Based on our results, 5-HT6R antagonist SB271046 can rescue the impaired LTP in chronic epileptic hippocampus, and this effect can be reversed by XE991, which selectively inhibits the M-current. Therefore, we speculate that the improved LTP by the antagonism of 5-HT6R may involve the regulation of M-channel.

In summary, the antagonism of 5-HT6R can decrease the excitability of the hippocampal pyramidal neurons in chronic epileptic rats by modulating the imbalance between excitatory and inhibitory neurotransmitters. The antagonism of 5-HT6R improves the impaired LTP in chronic epileptic rats by regulating the KCNQ2/3-mediated M-currents, especially KCNQ2-mediated M-currents, and may serve as a promising candidate in the clinical treatment of epilepsy.

## Data Availability Statement

The raw data supporting the conclusions of this article will be made available by the authors, without undue reservation, to any qualified researcher.

## Ethics Statement

The present study was approved by the Fujian Medical University Animal Experimental Ethical Committee (No. FJMUIACUC 2019-0058). The relevant experimental protocols and procedures followed the Guidelines for the Care and Use of Laboratory Animals in Fujian Medical University.

## Author Contributions

HH, WL, and CZ conceived and designed the study. CZ, RL, CL, MH, FL, GZ, YZ, and JM performed the experiments and analyzed the data. CZ wrote the paper. WL and HH revised the paper. All authors reviewed the results and approved the final version of the manuscript.

## Funding

This study was supported by the General Project of the National Natural Science Foundation of China (Grant No. 81671295 and No. 81901311), Fujian Province Natural Science Foundation Project (No. 2017J01198), and Joint Funds for the Innovation of Science and Technology, Fujian province (Grant number: 2018Y9014)

## Conflict of Interest

The authors declare that the research was conducted in the absence of any commercial or financial relationships that could be construed as a potential conflict of interest.
